# Dynamics of Bacterial and Fungal Communities and Metabolites During Aerobic Exposure in Whole-Plant Corn Silages With Two Different Moisture Levels

**DOI:** 10.3389/fmicb.2021.663895

**Published:** 2021-06-15

**Authors:** Chunsheng Bai, Chao Wang, Lin Sun, Haiwen Xu, Yun Jiang, Na Na, Guomei Yin, Sibo Liu, Yanlin Xue

**Affiliations:** ^1^Horticultural College, Shenyang Agricultural University, Shenyang, China; ^2^Inner Mongolia Engineering Research Center of Development and Utilization of Microbial Resources in Silage, Inner Mongolia Academy of Agriculture and Animal Husbandry Science, Hohhot, China; ^3^College of Foreign Languages, Inner Mongolia University of Finance and Economics, Hohhot, China; ^4^Department of Animal Sciences, University of Florida, Gainesville, FL, United States

**Keywords:** whole-plant corn silage, microbial communities, metabolites, aerobic stability, fermentation quality, fatty acids, amino acids

## Abstract

The study was aimed to investigate the effect of moisture content on microbial communities, metabolites, fermentation quality, and aerobic stability during aerobic exposure in whole-plant corn silages preserved long time to improve the quality and aerobic stability of the silage during feed-out. Corn plants with two different moisture levels (high-moisture content, 680 g/kg; low-moisture content, 620 g/kg) were harvested at one-third and two-thirds milk-line stages, respectively, ensiled in laboratory-scale silos, and then sampled at 350 day after ensiling and at 2 and 5 day after opening to investigate bacterial and fungal communities, metabolites, and aerobic stability. High-moisture content increased aerobic stability and pH and decreased lactic acid and microbial counts in silages (*P* < 0.05). During aerobic exposure, the low-moisture silages had higher pH and lactic acid bacterial count and lower lactic acid than the high-moisture silages (*P* < 0.05); *Acinetobacter* sp. was the most main bacterial species in the silages; *Candida glabrata* and unclassified *Candida* had an increasing abundance and negatively correlation with aerobic stability of high-moisture silages (*P* < 0.05), while *C. glabrata*, *Candida xylopsoci*, unclassified *Saccharomycetaceae*, and unclassified *Saccharomycetales* negative correlated with aerobic stability of low-moisture silages (*P* < 0.05) with a rising *Saccharomycetaceae*; the silages had a reducing concentration of total metabolites (*P* < 0.05). Moreover, the high-moisture silages contained greater total metabolites, saturated fatty acids (palmitic and stearic acid), essential fatty acids (linoleic acid), essential amino acids (phenylalanine), and non-essential amino acids (alanine, beta-alanine, and asparagine) than the low-moisture silages at 5 day of opening (*P* < 0.05). Thus, the high-moisture content improved the aerobic stability. *Acinetobacter* sp. and *Candida* sp. dominated the bacterial and fungal communities, respectively; *Candida* sp. resulted in the aerobic deterioration in high-moisture silages, while the combined activities of *Candida* sp. and *Saccharomycetaceae* sp. caused the aerobic deterioration in low-moisture silages. The greater aerobic stability contributed to preserve the palmitic acid, stearic acid, linoleic acid, phenylalanine, alanine, beta-alanine, and asparagine during aerobic exposure.

## Introduction

Corn is the most common crop for ensiling and the main forage source for ruminants worldwide because of the high biomass yield, suitable starch concentration, and good fermentation quality (Khan et al., 2015; Keshri et al., 2018; Guan et al., 2020; Zhang et al., 2020). To produce whole-plant corn silage, the plants are usually harvested between the one- and two-thirds milk-line stages (Xu et al., 2019), leading to differences in the moisture content of raw materials and in the fermentation quality and bacterial community of silages (Guan et al., 2018). However, the effects of different moisture content (high- and low-moisture content) or harvesting stages (one- and two-third milk-line stage) on the fungal community and metabolites in whole-plant corn silages remain unclear.

In the past 5 years, research in this area has primarily focused on the microbial communities in whole-plant corn silages. As the microbial community composition plays a crucial role in the fermentation quality and aerobic stability of silage, it is necessary to assess the bacterial and fungal communities to explain the different fermentation quality and aerobic stability among silages (Wang et al., 2021). Previous studies revealed the microbial community dynamics in whole-plant corn silages inoculated with lactic acid bacteria (LAB) (Keshri et al., 2018; Xu et al., 2020b) and derived from materials obtained from three areas in Iran (Gharechahi et al., 2017). Other recent investigations examined the bacterial community in whole-plant corn silages collected from five major ecological areas in southwestern China (Guan et al., 2018) and treated with additives (Romero et al., 2018; Guan et al., 2020; Zhang et al., 2020). During aerobic exposure, the fungus in silages becomes active and generates heat under aerobic conditions leading to aerobic deterioration and reduction of aerobic stability. Keshri et al. (2018) examined the bacterial and fungal communities of whole-plant corn silages at 5 day after aerobic exposure. Drouin et al. (2020) and Wang et al. (2021) revealed the fact that high fungal diversity can improve aerobic stability of whole-plant corn silage. However, little is known regarding bacterial and fungal community dynamics in whole-plant corn silages during aerobic exposure, especially at the species level. The PacBio single molecule in conjunction with real-time sequencing technology (SMRT) is a vital metagenomic approach that can cover the full read length of the DNA fragment multiple times, resulting in a reduced error rate and an increased ability to depict the microbial profile to species level precision (Schloss et al., 2016). The SMRT is considered suitable for precisely assessing the microbial community at the species level in silage (Guo et al., 2018; Xu et al., 2019, 2020a, b).

The silage metabolome is an area of recent research interest (Wilkinson and Muck, 2019). For example, studies have revealed the changes in metabolite contents of whole-plant corn silages and sainfoin silages treated with LAB inoculant (Xu et al., 2020a,b). Other studies investigated the metabolite profiles in whole-plant corn silages and alfalfa silages inoculated with LAB (Guo et al., 2018; Xu et al., 2019). Wu et al. (2020) revealed the distribution of metabolites in high-moisture sweet corn kernel silage. However, the metabolite dynamics in silages and the effects of aerobic stability on metabolite contents during aerobic exposure remain relatively uncharacterized.

The previous studies analyzed microbial communities and metabolites in silages for short- and medium-term storage (less than 150 day) (Guo et al., 2018; Xu et al., 2019, 2020a,b; Drouin et al., 2020; Wu et al., 2020), while the microbial communities and metabolites are little known in long-preserved silage (more than 300 day). Moreover, the whole-plant corn silage is usually prepared for supplying to ruminant throughout year. Thus, the objective of this study was to determine the bacterial and fungal communities as well as the metabolites of high- and low-moisture whole-plant corn silages after 350 day of ensiling (long-preserved silage) and their dynamics during aerobic exposure. Those could provide theoretical basis for regulation technique of quality and aerobic stability of whole-plant corn silage preserved for long time in silo. Our hypothesis was that the moisture content would influence the microbial communities, metabolites, and aerobic stability of whole-plant corn silages, and that the difference in aerobic stability would be associated with the differences in the bacterial and fungal communities and metabolites during aerobic exposure.

## Materials and Methods

### Materials and Silage Preparation

Corn (*Zea mays* L.) plants were grown on an experimental farm at Shenyang Agricultural University, Shenyang, China. Whole corn plants with high-moisture content (H; 680 g/kg) were harvested at the one-third milk-line stage from three corn fields (for three replicates) on September 10, 2018, whereas whole corn plants with low-moisture content (L; 620 g/kg) were harvested at the two-thirds milk-line stage from three other corn fields on the same day. The variety of corn was 23 Yu for ensiling (No. 2008022, Henan Dajingjiu Seed Industry Co., Ltd., Shangqiu, China). After harvesting, the fresh forage samples from the six fields were separately chopped into 1–2 cm pieces and then mixed uniformly. The chopped corn plant material from each field was randomly divided into four batches and ensiled in four polyethylene laboratory-scale silos (diameter, 20 cm; height, 30 cm) at a density of 750 kg/m^3^, respectively. The silos were stored at ambient temperature (22–25°C).

### Aerobic Stability Assessment and Sampling

The silos were opened after 350 day of ensiling to assess the aerobic stability as described by Ranjit and Kung, 2000 and Wang et al. (2020). For each field of high- and low-moisture silages, one silo selected randomly was used to measure the silages temperature and the ambient temperature by inserting a SMOWO Multi-Channel Data Logger (MDL-1048A; Shanghai Tianhe Automation Instrument Co., Ltd., Shanghai, China), and the other one selected randomly was sampled at 0 (H0 and L0), 2 (H2 and L2), and 5 (H5 and L5) d after opening for analyzing, respectively.

### Fermentation Quality

The silage samples were dried in a forced-air oven (BPG-9240A; Shanghai Yiheng Scientific Instrument Co., Ltd., Shanghai, China) at 65°C for 48 h to analyze the dry matter, which was ground through a 1 mm screen using a mill (FS-6D; Fichi Machinery Equipment Co., Ltd., Shandong, China). To prepare silage extracts, 25 g fresh silage was mixed with 225 mL sterile water and homogenized for 100 s using a flap type sterile homogenizer (JX-05; Shanghai Jingxin Industrial Development Co., Ltd., Shanghai, China), after which the homogenate was filtered through four layers of cheesecloth. The pH of the silage extracts was measured with a pH meter (PB-10; Sartorius, Göttingen, Germany). The organic acids (lactic acid, acetic acid, propionic acid, and butyric acid) concentrations in the silage extracts were determined by high-performance liquid chromatography (HPLC; 20A; Shimadzu Co., Ltd., Kyoto, Japan) with an SPD-20A diode array detector (210 nm) and a column (50°C; Shodex RS Pak KC-811; Showa Denko K.K., Kawasaki, Japan); the mobile phase was 3 mM HClO_4_ with 1.0 mL/min flow rate; the concentrations were obtained by comparing the curves of silage extracts with the standard curve of standard substances (Zhang et al., 2014). The ammonia nitrogen and the total nitrogen contents were measured with the Kjeltec autoanalyzer (8400; Foss Co., Ltd., Hillerød, Denmark) according to the Kjeldahl method.

### Bacterial and Fungal Communities

The LAB, *Escherichia coli*, aerobic bacteria, and yeast/mold counts in the silage extracts were determined by culturing on De Man Rogosa Sharpe agar, violet red bile agar, nutrient agar, and potato dextrose agar, respectively (Cai, 1999).

The total DNA was extracted from bacteria and fungi in the silage samples with a E.Z.N.A. ^®^Stool DNA Kit (D4015-04, Omega, Inc., United States). For SMRT sequencing, the full-length bacterial 16S rRNA genes were amplified by PCR with specific forward (5′-TAGRGTTYGATYMTGGCTCAG-3′) and reverse (5′-RGYTACCTTGTTACGACTT-3′) primers and the full-length fungal internal transcribed spacer (ITS) was amplified with specific forward (5′-TCCGTAGGTGAACCTGCGG-3′) and reverse (5′-TCCTCCGCTTATTGATATGC-3′) primers. The PCR program was as follows: 95°C for 3 min; 25 cycles of 98°C for 20 s, 57°C for 30 s, and 72°C for 90 s; 72°C for 2 min (Xu et al., 2019, 2020b). The 16S rRNA and ITS libraries were built with a Pacific Biosciences Template Prep Kit (Pacific Biosciences, Menlo Park, CA, United States) and then sequenced with the PacBio Sequel system (Pacific Biosciences). Raw circular consensus sequencing reads were obtained using the PacBio SMRT Link CCS software. The sequencing data were submitted to the NCBI Sequence Read Archive database (accession number: PRJNA661392).

### Metabolites

The fresh silage (5 g) and extraction liquid (10 mL; 70% methanol) were vortexed for 30 s, oscillated for 1 h at 4°C, and then filtered through a 0.22 μm membrane. Filtrates were dried to 1 mL by a vacuum concentrator in a glass vial for analysis (Xu et al., 2019; Yan et al., 2019). The silage extracts were analyzed using a liquid chromatography positive ion electrospray ionization tandem mass spectrometry (LC-ESIMS/MS) system (HPLC, Shim-pack UFLC SHIMADZU CBM30A system, Kyoto, Japan; MS, Applied Biosystems 4500 Q TRAP, Foster City, CA, United States). The conditions used for the analysis were as follows: HPLC column, Waters ACQUITY UPLC HSS T3 C18 (1.8 μm, 2.1 mm × 100 mm); mobile phase, water (0.04% acetic acid): acetonitrile (0.04% acetic acid); gradient program, 100:0 (v:v) at 0 min, 5:95 at 11.0 min, 5:95 at 12.0 min, 95:5 at 12.1 min, 95:5 at 15.0 min; flow rate, 0.40 mL/min; temperature, 40°C; injection volume, 5 μL. The effluent was then connected to an ESI-triple quadrupole-linear ion trap (QqQ-LIT) mass spectrometer (Xu et al., 2020b). The qualitative and quantitative analyses of metabolites as well as the pre-processing of the raw data were completed using the protocol developed by Yan et al. (2019). The relative concentrations of the detected metabolites were calculated as in Xu et al. (2019).

### Statistical Analyses

The data regarding the fermentation quality, aerobic stability and microbial counts were analyzed as a 2 × 3 factorial design. Model included 2 moisture contents, 3 aerobic exposure times and their interaction. The differences between 2 moisture contents, and among 3 sampling times were analyzed with the GLM procedure of SAS (SAS System for Windows, version 9.1.3; SAS Institute Inc., Cary, NC, United States). The interaction of moisture content and aerobic exposure time was analyzed using the PDIFF procedure of SAS. Correlations between the aerobic stability and the fungal community were analyzed using correlation analysis of the SAS software. The principal component analysis of metabolic profiles was analyzed using R 3.5.1 for Windows; the non-metric multi-dimensional scaling of microbial Bette diversity was analyzed by PRIMER 7 based on Bray-Curtis dissimilarities.

## Results

### Aerobic Stability and Fermentation Quality

**FIGURE 1:**
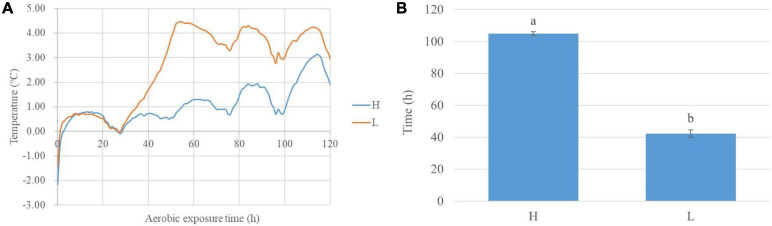
The degree of silage temperature (°C) above ambient temperature in whole-plant corn silage during aerobic exposure **(A)** and the time of silage temperature with 2°C above ambient temperature **(B)**. H, ensiled whole corn plants with a high moisture content (680 g/kg) harvested at the one-third milk-line stage; L, ensiled whole corn plants with a low moisture content (620 g/kg) harvested at the two-thirds milk-line stage.

During aerobic exposure, the time of high-moisture silages with 2°C above the ambient temperature was longer than low-moisture silages (*P* < 0.05) (Figure 1). The pH increased in high- and low-moisture silages (*P* < 0.05), while the low-moisture silages had a decreasing ammonia nitrogen (*P* < 0.05). Comparing with low-moisture silages, the high-moisture silages contained lower pH and greater lactic acid content at 0, 2, and 5 day (*P* < 0.05), and higher ammonia nitrogen at 5 day (*P* < 0.05). The moisture content affected (*P* < 0.05) the pH and the lactic and propionic acid concentrations. Additionally, the aerobic exposure time influenced (*P* < 0.05) the pH, propionic acid concentration, and ammonia nitrogen content, which were also interactionally affected (*P* < 0.05) by moisture content and aerobic exposure time (Table 1).

**TABLE 1:** pH, organic acid content (g/kg dry matter), ammonia nitrogen content (AN, g/kg total nitrogen), and microbial counts (log_10_ colony forming units/g fresh weight) of whole-plant corn silage during aerobic exposure.

Items	Treatments	Aerobic exposure time (d)			SEM^d^	*P*-value	Interaction		
					
		0	2	5			M^e^	T^e^	M*T^e^
pH	H^c^	3.50b^a^B^b^	3.47bB	3.75bA	0.014	< 0.001	< 0.0001	< 0.0001	< 0.0001
	L	3.66aB	3.64aB	4.19aA	0.023	< 0.001			
	SEM	0.003	0.016	0.028					
	*P*-value	<0.001	0.0015	0.0004					
Lactic acid	H	101.79a	103.60a	96.52a	5.415	0.6509	< 0.0001	0.2070	0.6190
	L	73.82b	67.21b	56.33b	6.186	0.2109			
	SEM	3.580	7.601	5.549					
	*P*-value	0.0052	0.0277	0.0069					
Acetic acid	H	15.79	15.65	13.59	2.107	0.7245	0.0119	0.7672	0.8739
	L	10.39	9.48	9.57	1.759	0.9224			
	SEM	1.725	2.450	1.524					
	*P*-value	0.0912	0.1493	0.1349					
Propionic acid	H	5.67aA	ND	ND	0.274	< 0.001	< 0.0001	< 0.0001	< 0.0001
	L	ND	ND	ND	−	−			
	SEM	0.335	−	−					
	*P*-value	0.0003	−	−					
Ammonia nitrogen	H	9.48	8.38	9.50a	0.730	0.5027	0.1046	0.0431	0.0239
	L	9.78A	8.61A	6.50bB	0.580	0.0192			
	SEM	0.394	0.974	0.446					
	*P*-value	0.6228	0.8755	0.0090					
Lactic acid bacteria	H	4.21bC	6.51bB	8.07bA	0.069	< 0.001	< 0.0001	< 0.0001	0.0010
	L	4.69aC	7.65aB	8.67aA	0.053	< 0.001			
	SEM	0.078	0.041	0.059					
	*P*-value	0.0133	<0.001	0.0020					
Aerobic bacteria	H	4.21bC	6.48bB	8.47A	0.066	< 0.001	0.0040	< 0.0001	0.0189
	L	5.02aC	7.15aB	8.46A	0.150	< 0.001			
	SEM	0.037	0.167	0.105					
	*P*-value	0.0001	0.0475	0.9495					
Yeasts	H	4.91bC	6.27bB	8.56A	0.195	< 0.001	0.0004	< 0.0001	0.0121
	L	5.33aC	7.44aB	8.54A	0.133	< 0.001			
	SEM	0.078	0.110	0.256					
	*P*-value	0.0189	0.0017	0.9586					

*^a^Values with different lowercase letters (a,b) indicate significant differences among treatments on the same day during aerobic exposure.*

*^b^Values with different uppercase letters (A, B, and C) indicate significant differences among days after opening for the same treatments (P < 0.05).*

*^c^H, ensiled whole corn plants with a high moisture content (680 g/kg) harvested at the one-third milk-line stage; L, ensiled whole corn plants with a low moisture content (620 g/kg) harvested at the two-thirds milk-line stage.*

*^d^SEM, standard error of means.*

*^e^M, moisture (silages with high or low moisture contents); T, aerobic exposure time; M × T, interaction between the moisture content and the aerobic exposure time.*

### Microbial Counts and Diversity

During aerobic exposure, the LAB, aerobic bacteria, and yeast counts increased in high- and low-moisture silages (*P* < 0.05). When compared with the high-moisture silages, the LAB, aerobic bacteria, and yeast was higher for L0 and L2 (*P* < 0.05) and LAB was higher for L5 (*P* < 0.05). An interaction effect (*P* < 0.05) was detected between the moisture content and aerobic exposure time on the LAB, aerobic bacteria, and yeast counts (*P* < 0.05). *Escherichia coli* and molds were not detected in silages (Table 1).

**FIGURE 2:**
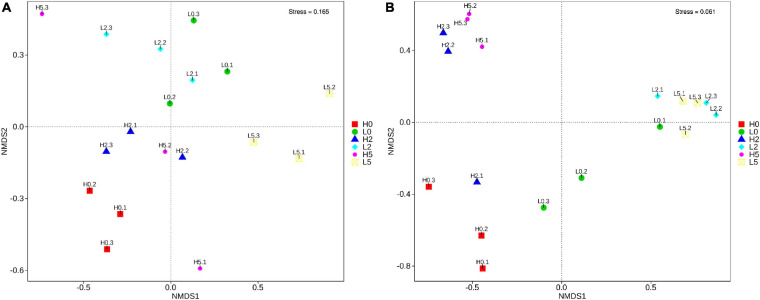
Non-metric multi-dimensional scaling based on Bray-Curtis dissimilarities in bacterial **(A)** and fungal **(B)** communities in whole-plant corn silages (*n* = 3). H, ensiled whole corn plants with a high moisture content (680 g/kg) harvested at the one-third milk-line stage; L, ensiled whole corn plants with a low moisture content (620 g/kg) harvested at the two-thirds milk-line stage.

The SMRT sequencing of the full-length 16S rRNA genes and ITS sequences generated 235,001 and 187,226 clean reads, respectively, for 18 whole-plant corn silage samples (Supplementary Table 1). The non-metric multi-dimensional scaling based on Bray-Curtis dissimilarities clearly separated the bacterial and fungal communities between the high- and low-moisture silages (Figure 2). In the high-moisture silages, the bacterial and fungal communities of H0 were clearly separated from those of H2 and H5. In the low-moisture silages, the bacterial community of L5 was separated from that of L0 and L2, whereas the fungal community of L0 was separated from that of L2 and L5 (Figure 2).

### Bacterial Community

**FIGURE 3:**
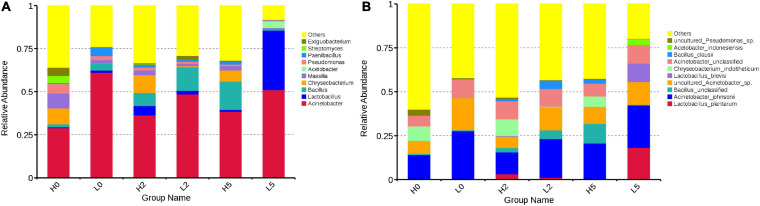
Relative abundance of bacterial communities in whole-plant corn silage **(A)**, at the genera level; **(B)**, at the species level). H, ensiled whole corn plants with a high moisture content (680 g/kg) harvested at the one-third milk-line stage; L, ensiled whole corn plants with a low moisture content (620 g/kg) harvested at the two-thirds milk-line stage.

At the genera level, *Acinetobacter* was the most main genus in H0 and L0, with abundances of 29.01 and 61.16%, respectively, followed by *Massilia* (8.59 and 1.83%, respectively), *Chryseobacterium* (9.31 and 0.145%, respectively), *Pseudomonas* (5.53 and 2.33%, respectively), *Bacillus* (1.71 and 4.10%, respectively), *Paenibacillus* (0.504 and 4.81%, respectively), *Exiguobacterium* (4.80 and 0.365%, respectively), *Streptomyces* (4.22 and 0.129%, respectively), and *Lactobacillus* (0.415 and 1.21%, respectively) (Figure 3A). During aerobic exposure, the high-moisture silages showed increased abundance of *Acinetobacter*, *Bacillus*, and *Paenibacillus*, but had the opposite effect on *Massilia*, *Pseudomonas*, and *Exiguobacterium*; moreover, *Lactobacillus* and *Chryseobacterium* increased at 2 day and decreased at 5 day after opening. The low-moisture silages showed increased abundance of *Lactobacillus* (2.04% for L2 and 34.53% for L5), but decreased abundance of *Massilia*, *Pseudomonas*, and *Paenibacillus*; *Acinetobacter* decreased in L2 (48.61%) and increased in L5 (51.03%), with the opposite dynamics observed for *Bacillus* and *Exiguobacterium* (Figure 3A).

At the species level, *Acinetobacter johnsonii*, *Chryseobacterium indoltheticum*, uncultured *Acinetobacter* sp., unclassified *Acinetobacter*, and uncultured *Pseudomonas* sp. were the main species in H0, with abundances of 13.83, 8.26, 7.37, 6.06, and 3.53%, respectively. *Acinetobacter johnsonii* (27.37%), uncultured *Acinetobacter* sp. (18.27%), and unclassified *Acinetobacter* (10.46%) were the main species in L0. *Lactiplantibacillus plantarum* (formerly *Lactobacillus plantarum*) and *Levilactobacillus brevis* (formerly *Lactobacillus brevis*) were minor taxa in H0 (0.30 and 0.31%, respectively) and L0 (0.01 and 0.14%, respectively) (Figure 3B). During aerobic exposure, the abundances of *A. johnsonii*, uncultured *Acinetobacter* sp., and unclassified *Acinetobacter* exceeded 6.05% in the high- and low-moisture silages (Figure 3B).

### Fungal Community

**FIGURE 4:**
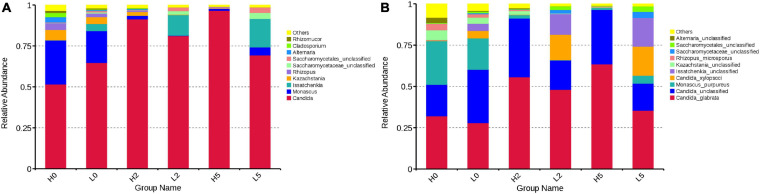
Relative abundance of fungal communities in whole-plant corn silage **(A)**, at the genera level; **(B)**, at the species level. H, ensiled whole corn plants with a high moisture content (680 g/kg) harvested at the one-third milk-line stage; L, ensiled whole corn plants with a low moisture content (620 g/kg) harvested at the two-thirds milk-line stage.

At the genera level, *Candida* and *Monascus* were the predominant fungal genera in H0 (57.64 and 26.81%, respectively) and L0 (64.74 and 19.43%, respectively), followed by *Kazachstania*, *Rhizopus*, *Alternaria*, *Cladosporium*, and *Rhizomucor* in H0 and *Issatchenkia*, *Kazachstania*, and *Rhizopus* in L0 (more than 1% of abundance) (Figure 4A). At the species level, the dominant fungal species in H0 and L0 were *Candida glabrata* (32.11 and 27.91%, respectively), unclassified *Candida* (19.08 and 32.27%, respectively), and *Monascus purpureus* (26.38 and 19.02%, respectively), followed by unclassified *Kazachstania*, *Rhizopus microsporus*, and unclassified *Alternaria* in H0 and *Candida xylopsoci*, unclassified *Issatchenkia*, unclassified *Kazachstania*, and *R. microsporus* in L0 (more than 1% of abundance) (Figure 4B).

During aerobic exposure, in high-moisture silages, the abundance of *Candida* increased to 96.61% in H5, whereas the abundance of *Monascus*, *Kazachstania*, and *Rhizopus* decreased (Figure 4A). The abundance of *C. glabrata*, as main fungal species, increased to 63.50% in H5, whereas the abundance of unclassified *Candida* increased in H2 (35.50%) and then decreased in H5 (32.99%) (Figure 4B). The degree of silage temperature (°C) above ambient temperature (DSTAT) was positively correlated with *Candida*, *C. glabrata*, and unclassified *Candida* abundances (Table 2). In low-moisture silages, the abundance of *Candida* increased in L2 (81.16%) and decreased in L5 (69.40%) (Figure 4A). More specifically, the abundance of *C. glabrata* was 48.05 and 35.41% in L2 and L5, respectively, whereas the abundance of *C. xylopsoci* increased to 17.60% in L5 (Figure 4B). The DSTAT was positively correlated with the abundance of *Candida*, *Issatchenkia*, unclassified *Saccharomycetaceae*, unclassified *Saccharomycetales*, *C. glabrata*, and *C. xylopsoci* (Table 2).

**TABLE 2:** Correlations between the degree of silage temperature (°C) above ambient temperature (DSTAT) and the fungal community in whole-plant corn silages.

Fungal community^a^	DSTAT of silages with high-moisture	DSTAT of silages with low-moisture
Yeast counts	0.98491***^b^	0.88214**
*Candida*	0.78644*	0.44438
*Issatchenkia*	−	0.81445**
*Saccharomycetaceae* unclassified	−	0.70414*
*Saccharomycetales* unclassified	−	0.69807*
*Candida glabrata*	0.88522**	0.63390
*Candida* unclassified	0.56591	−
*Candida xylopsoci*	−	0.71483*

*^a^Correlation coefficients between the fungal communities (at the genera level, top 10; at the species level, top 10) and the DSTAT of high- and low-moisture whole-plant corn silages > 0.*

^b^*P < 0.05, P ≥ 0.01; **P < 0.01, P ≥ 0.001; ***P < 0.001.

### Metabolites

**FIGURE 5:**
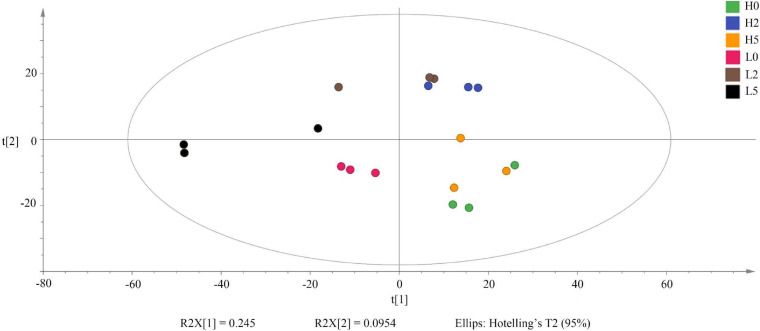
Principal component analysis of metabolites in whole-plant corn silages (*n* = 3). H, ensiled whole corn plants with a high moisture content (680 g/kg) harvested at the one-third milk-line stage; L, ensiled whole corn plants with a low moisture content (620 g/kg) harvested at the two-thirds milk-line stage.

A total of 668 substances were detected in 18 samples of high- and low-moisture whole-plant corn silages, of which 288 substances were identified (Supplementary Table 2). The high- and low-moisture silages were clearly separated based on principal component analysis of the metabolomes; the silages at 5 day after opening were clearly separated from the silages at 0 and 2 days after opening (Figure 5).

The H5 and L5 contained lower total metabolites, total essential amino acids, and total non-essential amino acids than H0 and H2, and L0 and L2, respectively (*P* < 0.05); the concentrations of total saturated fatty acids and total essential fatty acids in L5 were lower those in L0 and L2 (*P* < 0.05). Moreover, comparing with L5, the H5 had higher total metabolites, total saturated fatty acids, total essential fatty acids, total essential amino acids, and total non-essential amino acids (*P* < 0.05), which were affected by moisture content and aerobic exposure time (*P* < 0.05) (Table 3).

**TABLE 3:** Fold-change concentrations (log_2_ relative concentrations) of total metabolites, saturated fatty acids, essential fatty acids, essential amino acids and non-essential amino acids in whole-plant corn silages during aerobic exposure.

Items	Treatments	Aerobic exposure time (d)			SEM^d^	*P*-value	Interaction		
					
		0	2	5			M^e^	T	M*T
Total metabolites	H^c^	12.0A	12.5A	10.1a^a^B^b^	0.4429	0.0197	0.0101	0.0002	0.1485
	L	11.88A	11.03A	8.03bB	0.4623	0.0025			
	SEM	0.5032	0.4288	0.4213					
	*P*-value	0.9013	0.0735	0.0257					
Total saturated fatty acids	H	6.60	7.03	5.25a	0.4767	0.0861	0.0068	0.0004	0.1265
	L	6.49A	5.65A	3.33bB	0.3021	0.0008			
	SEM	0.4482	0.4626	0.2508					
	*P*-value	0.8707	0.1032	0.0058					
Total essential fatty acids	H	3.90	4.24	2.31a	0.5446	0.0950	0.0025	0.0004	0.1743
	L	3.26A	2.81A	−0.25bB	0.5113	0.0056			
	SEM	0.5174	0.5085	0.5575					
	*P*-value	0.4312	0.1183	0.0316					
Total essential amino acids	H	8.76A	9.31A	6.87aB	0.4398	0.0178	0.0122	0.0002	0.0691
	L	8.82A	7.98A	3.95bB	0.6485	0.0039			
	SEM	0.4938	0.4174	0.7091					
	*P*-value	0.9357	0.0974	0.0439					
Total non-essential amino acids	H	9.97A	10.4A	7.93aB	0.4318	0.0137	0.0194	0.0002	0.1419
	L	10.1A	8.96A	5.84bB	0.6014	0.0063			
	SEM	0.4878	0.5621	0.5181					
	*P*-value	0.9133	0.1368	0.0465					

*^a^Values with different lowercase letters (a,b) indicate significant differences among treatments on the same day during aerobic exposure.*

*^b^Values with different uppercase letters (A, B, and C) indicate significant differences among days after opening for the same treatments (P < 0.05).*

*^c^H, ensiled whole corn plants with a high moisture content (680 g/kg) harvested at the one-third milk-line stage; L, ensiled whole corn plants with a low moisture content (620 g/kg) harvested at the two-thirds milk-line stage.*

*^d^SEM, standard error of means.*

*^e^M, moisture (silages with high or low moisture contents); T, aerobic exposure time; M × T, interaction between the moisture content and the aerobic exposure time.*

## Discussion

The whole-plant corn silage has become a predominant forage for dairy industry worldwide (Ferraretto et al., 2018) and is produced for feeding dairy cow throughout year. However, the previous studies mainly focused on the fermentation quality, microbial communities, metabolites, and aerobic stability in whole-plant corn silage with single moisture content for short- and medium-term preservation (less than 150 day) (Guo et al., 2018; Xu et al., 2019, 2020a,b; Drouin et al., 2020; Wu et al., 2020). In the present study, the bacterial and fungal communities, metabolites, and aerobic stability of high- and low-moisture whole-plant corn silage preserved for long-term (350 day) was studied, which can help to regulate quality of whole-plant corn silage packed in end of silo.

The high- and low-moisture silages had similar dynamics of DSTAT in the 28 h (Figure 1A); moreover, the DSTAT of low-moisture silage began to increase and was 2°C at 42 h, and the DSTAT of high-moisture silage was 2°C at 106 h (Figure 1B). Indicated that the high-moisture silage had great aerobic stability. Those might be resulted from that, during aerobic exposure after 350 day of ensiling, the high-moisture silages had lower pH and higher lactic acid content than the low-moisture silages, because of the inhibitory effects of the acidic environment on some undesirable microbes in silages (Ávila and Carvalho, 2019). Those are consistent with Schmidt and Kung (2010), who found that aerobic stability was greater for whole-plant corn silages with 67.6% moisture than for silages with 61.8% moisture (60 h vs. 42 h).

For high- and low-moisture silages at 350 day after ensiling, the acetic acid content was less 30 g/kg DM, and the butyric acid was not detected. According to the evaluation system for fermentation quality of silages from contents of butyric and acetic acids (Kaiser and Weiss, 2005), the score of the silages was 100 and their mark was first. Indicated that the high- and low-moisture silages had satisfactory fermentation quality. During aerobic exposure, the microbial counts increased in both high- and low-moisture silages (Table 1), showed that the LAB, aerobic bacteria, and yeasts began to proliferate and activate violently under aerobic and acidic condition, because LAB and yeasts are facultatively anaerobic microorganism. Those might result in no difference between at 0 and 2 day after opening in pH and lactic acid of high- and low-moisture silages (Table 1). Drouin et al. (2020) also reported that the pH and lactic acid were similar in whole-plant corn silage with LAB inoculants at 0 and 2 d after aerobic exposure. Moreover, in the present study, the low-moisture silage had greater yeast count than the high-moisture silage (Table 1), which might one of reasons leading to poor aerobic stability.

The whole-plant corn silage is a microbial fermentation product, analyzing the bacterial and fungal communities is helping to understand the different fermentation quality and aerobic stability among silages (Wang et al., 2021). The key microorganisms are *Lactobacillus* group during fermentation process in whole-plant corn silage (Sun et al., 2021). In the present study, however, *Acinetobacter* was the predominant bacterial genus in the high- and low-moisture silages after ensiling for 350 d, and *Lactobacillus* was a minor taxon (Figure 3A). Previous studies showed that *Lactobacillus* dominated bacterial community in whole-plant corn silage after ensiling for 32, 60, and 90 day (Gharechahi et al., 2017; Guan et al., 2018, 2020; Keshri et al., 2018; Romero et al., 2018; Xu et al., 2019, 2020a; Zhang et al., 2020) and *Acinetobacter* was a minor taxon in whole-plant corn silage (Keshri et al., 2018; Xu et al., 2019; Zhang et al., 2020). The differences in the bacterial community might be resulted from the storage times (350 day vs. 32, 60, and 90 day). The effects of long- and short-term storage on the silage bacterial community need further investigation. In the present study, the epiphytic *Lactobacillus* on the whole-plant corn before ensiling might be less resistant to acidic environments (pH < 4.0) than *Acinetobacter*, resulting in lower abundance of *Lactobacillus* in whole-plant corn silage stored for long periods. Sun et al. (2021) reported that *Lactobacillus* dominated the bacterial community after 2 day of ensiling and had reducing abundance during stable phase in whole-plant corn silage with low pH (<4.0). Thus, the *Lactobacillus* might also control the middle prophase of fermentation process, which contributed to the high lactic acid content in high- and low-moisture silages at 350 day after ensiling (101.79 and 73.82 g/kg DM, respectively). During aerobic exposure, *Acinetobacter* were the dominant bacteria in high- and low-moisture silages, whereas *Lactobacillus* was one of the dominant genera in L5 and a minor taxon in the high-moisture silages (Figure 3A). However, Keshri et al. (2018) identified *Lactobacillus* as the dominant bacteria and *Acinetobacter* as a minor taxon in whole-plant corn silages after a 5-day aerobic exposure (95.50% vs. 0.03%). The abundance of *Acinetobacter* was greater in the low-moisture silages with high pH, low organic acid content (Table 1), and limited aerobic stability (Figure 1) than that in H2 and H5 (Figure 3A). This was consistent with the findings of Liu et al. (2019), who detected *Acinetobacter* as one of the dominant bacteria in barley silages with high pH after 5 and 7 day of aerobic exposure. The abundance of *Bacillus* increased in the high-moisture silages during aerobic exposures but increased in L2 and decreased in L5. These results were similar to those of a study by Wang et al. (2020), which revealed that the abundance of *Bacillus* species increased in sugarcane top silages after aerobic spoilage.

The predominant *Acinetobacter* species were *A. johnsonii*, uncultured *Acinetobacter* sp., and unclassified *Acinetobacter*, whereas *L. plantarum* and *L. brevis* were the main LAB species in whole-plant corn silages after ensiling for 350 day (Figure 3B). Xu et al. (2019, 2020b), using SMRT sequencing, revealed that *L. parafarraginis*, *L. silage*, *L. buchneri*, *L. farciminis*, and *L. paralimentarius* were the main species and predominant bacterial community in whole-plant corn silage after ensiling for 90 day. *Acinetobacter* species are a group of Gram-negative, strictly aerobic, non-fermenting organisms that are ubiquitous in polluted aquatic environments, sewage, vegetables, and can be pathogenic in animals and humans (Xia et al., 2008; Malick et al., 2020). In the present study, they might have been present in the materials before ensiling, have greater acid resistance than other bacteria in silages and therefore dominated the bacterial community during aerobic exposure (Figure 3B). *Acinetobacter* species might proliferate rapidly in an acidic and aerobic environment and result to the aerobic deterioration of silages (Liu et al., 2019). Those above-mentioned suggested that it is necessary ensiling whole-plant corn with inoculants that has greater capacity of acid production and resistance, especially *Lactobacillus* inhibiting undesired microorganism. Sanders and Lebeer (2020) and Zheng et al. (2020) reported the reclassification of genus *Lactobacillus* into 25 genera (*Lactobacillus delbrueck ii* group, *Paralactobacillus* and 23 novel genera) according to the core genome phylogeny, the (conserved) pairwise average amino acid identity, the clade-specific signature genes, the physiological criteria and the ecology of the organisms. The new names of the bacterial species in the present study were *Lactiplantibacillus plantarum* (formerly *Lactobacillus plantarum*) and *Levilactobacillus brevis* (formerly *Lactobacillus brevis*).

*Candida* and *Monascus* were the dominant fungal genera in high- and low-moisture silages after ensiling for 350 day (Figure 4A). This was consistent with the findings of Keshri et al. (2018), who showed that *Candida* and *Monascus* dominated the fungal community of whole-plant corn silages after 90 day of ensiling. Previous studies had identified the dominant fungal genera as *Debaryomycetaceae*, *Pichiaceae*, and *Saccharomycetales* incertae sedis in whole-plant corn silages at 100 day (Romero et al., 2018), *Kazachstania*, *Pichia*, and *Cryptococcus* in small grain silages at 90 day (Duniere et al., 2017), *Issatchenkia* in barley silages at 60 day (Liu et al., 2019), and *Candida*, *Kazachstania*, and *Pichia* in sugarcane top silages (Wang et al., 2020). At the species level, the predominant fungal species in the high- and low-moisture silages were *C. glabrata*, *C. xylopsoci*, unclassified *Candida*, *M. purpureus*, unclassified *Issatchenkia*, unclassified *Kazachstania*, and *R. microsporus* (Figure 4B). Wang et al. (2020) detected *Kazachstania humilis*, *C. ethanolica*, and unclassified *Pichia* as the main fungal species in sugarcane top silages lacking additives. The difference in fungal community among studies might be related to differences in the original epiphytic fungi in raw materials, in the fermentation process, or in the fungal community of soil (Keshri et al., 2018). *Candida glabrata*, which is a human fungal pathogen, has been isolated in baled grass silage (O’Brien et al., 2007), but there have been no reports describing the detection of *C. xylopsoci*, *M. purpureus*, and *R. microsporus* in silages.

During aerobic exposure, *Candida* predominated the fungal community and *C. glabrata* was the dominant fungal species in silages (Figure 4B). In the high-moisture silages, the dominant species, *C. glabrata* and unclassified *Candida*, enhanced the aerobic deterioration of high-moisture silages during aerobic exposure, as reflected by the positive correlation of DSTAT with *C. glabrata* and unclassified *Candida* (Table 2). This is consistent with the findings of Wang et al. (2020), who revealed that *Candida* is the fungal genus most associated with the aerobic deterioration of sugarcane top silages. However, Liu et al. (2019) observed that *Issatchenkia* species dominated the fungal community of barley silages after aerobic exposure. In the low-moisture silages, the poor aerobic stability might have been the result of the combined effects of some fungal populations because of the positive correlation between the DSTAT and the abundances of *Candida*, *Issatchenkia*, unclassified *Saccharomycetaceae*, unclassified *Saccharomycetales*, *C. glabrata*, and *C. xylopsoci* (Table 2). Previous studies revealed aerobic deterioration by the combined activities of microbes, including *Candida* and *Monascus* species in whole-plant corn silages (Keshri et al., 2018), *Kazachstania*, *Corollospora*, and *Pichia* species in small grain silages (Duniere et al., 2017), *Pichia* and *Wickerhamomyces* species in sugarcane top silages (Zhang et al., 2019), and *Kazachstania* and *Pichia* species in sugarcane top silages (Wang et al., 2020).

The high-moisture silages with better aerobic stability contained higher relative concentrations of 3, 35, and 127 identified metabolites than low-moisture silages at 0, 2, and 5 d after opening, respectively (Figure 1 and Supplementary Tables 3A–C), and had greater total metabolites at 5 day (Table 3). Moreover, in high- and low-moisture silages, the top 10 bacterial species (except for *Chryseobacterium indoltheticum*) did not correlate with 285 in 292 identified metabolites and total metabolites, and the top 3 fungal species (accounted for 78.39% of average abundance in fungal community) also did not have correlation with 283 of 292 identified metabolites and total metabolites (Supplementary Figure 1). Those mentioned-above suggested that during aerobic exposure, the satisfactory aerobic stability rather than microbial communities might help to preserve metabolites in whole-plant corn silages. Earlier studies indicated the inoculant increased the relative concentrations of some metabolites (e.g., organic acids, amino acids, and fatty acids) in whole-plant corn silages, sainfoin silages, and alfalfa silages with good fermentation quality (Guo et al., 2018; Xu et al., 2020a,b). In the present study, the silages at 5 day after opening contained lower total metabolites than at 0 and 2 day (Table 3), and the DSTAT had negative correlation with 276 in 292 identified metabolites (Supplementary Table 3D), which indicated that the reducing metabolites might be resulted from the rising temperature of silage during aerobic exposure. In the present study, the H5 contained less saturated fatty acids (palmitic and stearic acid), essential fatty acids (linoleic acid), essential amino acids (phenylalanine), and non-essential amino acids (alanine, beta-alanine, and asparagine) than L5 (Table 3 and Supplementary Table 4); moreover, the high-moisture silages had greater aerobic stability (Figure 1). Those implied that the greater aerobic stability contributed to the preservation of the palmitic acid, stearic acid, linoleic acid, phenylalanine, alanine, beta-alanine, and asparagine during aerobic exposure. It is necessary to improve the aerobic stability of whole-plant corn silage during feed-out for preserving those metabolites mentioned above. Palmitic and stearic acids as saturated fatty acids are commonly used for fatty acid supplementation to increase milk yield and milk components (Souza et al., 2018; Shepardson et al., 2020; Western et al., 2020). These fatty acids are mainly distributed in the kernels and leaves of corn plants (Baldin et al., 2018). In addition, the linoleic, linolenic, and arachidonic acids are essential fatty acids that cannot be synthesized by metabolic pathways in mammals and must be obtained from food (Vogel et al., 2020). Animal feed rich in linoleic and linolenic acids can improve the ruminal synthesis of conjugated linoleic acid isomers, some of which have health-promoting effects in mammals (Haubold et al., 2020). In corn plants, linoleic acid is mainly localized in kernels and linolenic acid is primarily distributed in kernels and leaves (Baldin et al., 2018). The essential amino acids (valine, phenylalanine, isoleucine, methionine, and threonine) were detected in the present study. Previous investigations also detected essential amino acids in whole-plant corn silages (lysine, methionine, and phenylalanine) (Xu et al., 2020b) and in alfalfa silages (threonine and valine) (Guo et al., 2018).

In summary, the high-moisture whole-plant corn silages exhibited greater aerobic stability than the low-moisture silages. During aerobic exposure, *A. johnsonii*, *L. plantarum*, unclassified *Bacillus*, and uncultured *Acinetobacter* sp. were the main bacterial species. The fungal community mainly comprised *C. glabrata*, and unclassified *Candida* species, which were associated with the aerobic deterioration in the high-moisture silage. *Candida glabrata*, *C. xylopsoci*, unclassified *Issatchenkia*, and unclassified *Kazachstania* decreased the aerobic stability of the low-moisture silages. The greater aerobic stability could help to preserve the metabolites, palmitic acid, stearic acid, linoleic acid, phenylalanine, alanine, beta-alanine, and asparagine during aerobic exposure.

## Data Availability Statement

The datasets presented in this study can be found in online repositories. The names of the repository/repositories and accession number(s) can be found in the article/Supplementary Material.

## Author Contributions

CB, CW, and YX designed the study, wrote the manuscript, and analyzed the data. CB, CW, LS, HX, NN, YJ, and GY performed the experiments. HX, YJ, and YX reviewed and edited the manuscript. YX funded and supervised the experiments. All authors reviewed the manuscript.

## Conflict of Interest

The authors declare that the research was conducted in the absence of any commercial or financial relationships that could be construed as a potential conflict of interest.

## Supplementary Material

Supplementary Figure 1 Pearson correlation heatmap of top 10 species of bacterial and fungal communities with total metabolites and identified metabolites. The relative concentration of metabolites increasing from top to bottom of the figure. ^∗^*p* < 0.05; ^∗∗^*p* < 0.01.

Supplementary Table 1 Reads of 16S rRNA genes and ITS sequences in whole-plant corn silage. H, ensiled whole corn plants with a high moisture content (680 g/kg) harvested at the one-third milk-line stage; L, ensiled whole corn plants with a low moisture content (620 g/kg) harvested at the two-thirds milk-line stage.

Supplementary Table 2 Relative concentration of metabolites detected in whole-plant corn silages. H, ensiled whole corn plants with a high moisture content (680 g/kg) harvested at the one-third milk-line stage; L, ensiled whole corn plants with a low moisture content (620 g/kg) harvested at the two-thirds milk-line stage.

Supplementary Table 3 Different identified metabolites between H0 and L0 **(A)**, H2 and L2 **(B)**, and H5 and L5 **(C)** correlation between the degree of silage temperature above ambient temperature (DSTAT) and the identified metabolites **(D)**. H, ensiled whole corn plants with a high moisture content (680 g/kg) harvested at the one-third milk-line stage; L, ensiled whole corn plants with a low moisture content (620 g/kg) harvested at the two-thirds milk-line stage.

Supplementary Table 4 Fold-change concentrations (log_2_ relative concentrations) of fatty acid and amino acid in whole-plant corn silages during aerobic exposure. H, ensiled whole corn plants with a high moisture content (680 g/kg) harvested at the one-third milk-line stage; L, ensiled whole corn plants with a low moisture content (620 g/kg) harvested at the two-thirds milk-line stage.
