# Supplementary figures and images for: Dynamics of Bacterial and Fungal Communities and Metabolites During Aerobic Exposure in Whole-Plant Corn Silages With Two Different Moisture Levels
Source: Front Microbiol. 2021 Jun 15;12:663895. doi: 10.3389/fmicb.2021.663895 (PMC8239417; doi:10.3389/fmicb.2021.663895)

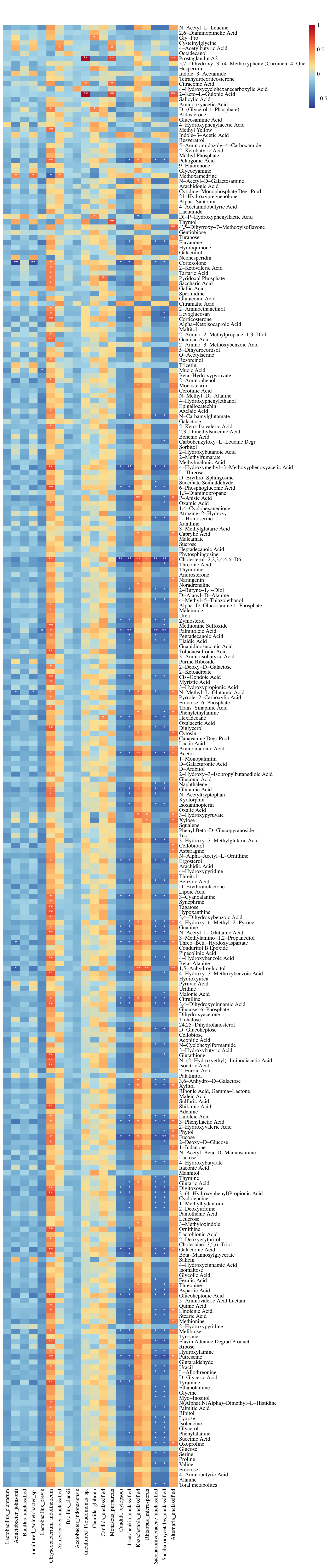

Supplement: Supplementary Figure 1 — Pearson correlation heatmap of top 10 species of bacterial and fungal communities with total metabolites and identified metabolites. The relative concentration of metabolites increasing from top to bottom of the figure. ∗p < 0.05; ∗∗p < 0.01. [file Data_Sheet_1.PDF]
