# Supplementary material for: Dynamics of Bacterial and Fungal Communities and Metabolites During Aerobic Exposure in Whole-Plant Corn Silages With Two Different Moisture Levels
Source: Front Microbiol. 2021 Jun 15;12:663895. doi: 10.3389/fmicb.2021.663895 (PMC8239417; doi:10.3389/fmicb.2021.663895)
Supplement: Supplementary Table 1 — Reads of 16S rRNA genes and ITS sequences in whole-plant corn silage. H, ensiled whole corn plants with a high moisture content (680 g/kg) harvested at the one-third milk-line stage; L, ensiled whole corn plants with a low moisture content (620 g/kg) harvested at the two-thirds milk-line stage. [file Table_1.DOCX]

| Table S1 Reads of 16S rRNA genes and ITS sequences in whole-plant corn silage | | | | | |
| --- | --- | --- | --- | --- | --- |
| Sample | 16S rRNA genes | |  | ITS sequences | |
|  | Raw reads | Clean reads |  | Raw reads | Clean reads |
| H0-1 | 13856 | 11573 |  | 9016 | 8615 |
| H0-2 | 14393 | 10988 |  | 9935 | 8737 |
| H0-3 | 10232 | 8064 |  | 10579 | 8561 |
| L0-1 | 20672 | 18350 |  | 10126 | 9541 |
| L0-2 | 20524 | 19406 |  | 9668 | 9382 |
| L0-3 | 20545 | 19766 |  | 11053 | 9632 |
| H2-1 | 12388 | 10538 |  | 12096 | 7534 |
| H2-2 | 19222 | 17375 |  | 7892 | 7786 |
| H2-3 | 11593 | 9641 |  | 10215 | 10040 |
| L2-1 | 14036 | 12081 |  | 11839 | 11499 |
| L2-2 | 12369 | 10739 |  | 9346 | 8792 |
| L2-3 | 12627 | 11123 |  | 10366 | 9748 |
| H5-1 | 15909 | 13475 |  | 17932 | 17458 |
| H5-2 | 14355 | 12533 |  | 11810 | 11701 |
| H5-3 | 18028 | 15082 |  | 13438 | 13216 |
| L5-1 | 12447 | 10535 |  | 9831 | 9276 |
| L5-2 | 12826 | 11344 |  | 18399 | 17537 |
| L5-3 | 13680 | 12388 |  | 8660 | 8171 |
| Total | 269702 | 235001 |  | 202201 | 187226 |

H, ensiled whole corn plants with a high moisture content (680 g/kg) harvested at the one-third milk-line stage; L, ensiled whole corn plants with a low moisture content (630 g/kg) harvested at the two-thirds milk-line stage.
