# Supplementary material for: Dynamics of Bacterial and Fungal Communities and Metabolites During Aerobic Exposure in Whole-Plant Corn Silages With Two Different Moisture Levels
Source: Front Microbiol. 2021 Jun 15;12:663895. doi: 10.3389/fmicb.2021.663895 (PMC8239417; doi:10.3389/fmicb.2021.663895)
Supplement: Supplementary Table 4 — Fold-change concentrations (log2 relative concentrations) of fatty acid and amino acid in whole-plant corn silages during aerobic exposure. H, ensiled whole corn plants with a high moisture content (680 g/kg) harvested at the one-third milk-line stage; L, ensiled whole corn plants with a low moisture content (620 g/kg) harvested at the two-thirds milk-line stage. [file Table_4.DOCX]

**Table S4** Fold-change concentrations (log2 relative concentrations) of fatty acid and amino acid in whole-plant corn silages during aerobic exposure

| Items | | Treatments | Aerobic exposure time (d) | | | SEM^4^ | *P*-value | Interaction | | |
| --- | --- | --- | --- | --- | --- | --- | --- | --- | --- | --- |
|  |  |  | 0 | 2 | 5 |  |  | M^5^ | T | M*T |
| Saturated fatty acids | Palmitic acid | H^3^ | 6.38 | 6.82 | 5.07a^1^ | 0.4755 | 0.0919 | 0.0064 | 0.0005 | 0.1260 |
|  |  | L | 6.26A | 5.43A | 3.14Bb^2^ | 0.3122 | 0.0010 |  |  |  |
|  |  | SEM | 0.4548 | 0.4557 | 0.2658 |  |  |  |  |  |
|  |  | *P*-value | 0.8577 | 0.0980 | 0.0069 |  |  |  |  |  |
|  | Stearic acid | H | 3.68A | 4.07A | 2.00aB | 0.4827 | 0.0491 | 0.0107 | 0.0002 | 0.1277 |
|  |  | L | 3.67A | 2.71B | 0.201bC | 0.2628 | 0.0002 |  |  |  |
|  |  | SEM | 0.4012 | 0.5016 | 0.2008 |  |  |  |  |  |
|  |  | *P*-value | 0.9881 | 0.1274 | 0.0032 |  |  |  |  |  |
|  | Myristic acid | H | -1.21 | -0.99 | -2.17 | 0.8035 | 0.5744 | 0.1737 | 0.1697 | 0.3275 |
|  |  | L | -1.6 | -2.08 | -9.84 | 3.3430 | 0.2267 |  |  |  |
|  |  | SEM | 0.8397 | 0.5349 | 4.0914 |  |  |  |  |  |
|  |  | *P*-value | 0.7703 | 0.2254 | 0.2556 |  |  |  |  |  |
|  | Caprylic acid | H | -1.37 | -11.9 | -9.00 | 4.540 | 0.3095 | 0.4158 | 0.1121 | 0.3313 |
|  |  | L | -1.32 | -2.67 | -10.6 | 3.1682 | 0.1631 |  |  |  |
|  |  | SEM | 0.4586 | 4.169 | 5.326 |  |  |  |  |  |
|  |  | *P*-value | 0.9384 | 0.1927 | 0.8443 |  |  |  |  |  |
| Essential fatty acids | Linolenic acid | H | 3.64 | 3.91 | 1.95 | 0.5695 | 0.0995 | 0.1389 | 0.1229 | 0.3335 |
|  |  | L | 2.78 | 2.36 | -7.15 | 4.1468 | 0.2390 |  |  |  |
|  |  | SEM | 0.5521 | 0.5613 | 5.065 |  |  |  |  |  |
|  |  | *P*-value | 0.3335 | 0.1239 | 0.2730 |  |  |  |  |  |
|  | Linoleic acid | H | 1.21 | 1.95 | 0.025a | 0.4872 | 0.0802 | 0.0037 | < 0.0001 | 0.0214 |
|  |  | L | 1.40A | 0.694A | -2.23bB | 0.3054 | 0.0003 |  |  |  |
|  |  | SEM | 0.4065 | 0.4415 | 0.3683 |  |  |  |  |  |
|  |  | *P*-value | 0.7605 | 0.1158 | 0.0123 |  |  |  |  |  |
|  | Arachidonic acid | H | -8.15 | -17.3b | -5.67 | 3.0163 | 0.0738 | 0.8724 | 0.4227 | 0.0131 |
|  |  | L | -17.8 | -3.58a | -11.1 | 3.2333 | 0.0556 |  |  |  |
|  |  | SEM | 3.662 | 0.8230 | 3.903 |  |  |  |  |  |
|  |  | *P*-value | 0.1352 | 0.0003 | 0.3801 |  |  |  |  |  |
| Essential amino acids | Valine | H | 7.44A | 8.05A | 5.68B | 0.4731 | 0.0289 | 0.0478 | 0.0160 | 0.0454 |
|  |  | L | 7.64A | 6.74A | -12.9B | 5.0815 | 0.0485 |  |  |  |
|  |  | SEM | 0.5238 | 0.4762 | 6.210 |  |  |  |  |  |
|  |  | *P*-value | 0.8041 | 0.1238 | 0.1017 |  |  |  |  |  |
|  | Phenylalanine | H | 6.95A | 7.47aA | 5.03aB | 0.4372 | 0.0171 | 0.0101 | < 0.0001 | 0.1699 |
|  |  | L | 6.82A | 6.21bA | 3.25bB | 0.3250 | 0.0005 |  |  |  |
|  |  | SEM | 0.4953 | 0.2501 | 0.3704 |  |  |  |  |  |
|  |  | *P*-value | 0.8632 | 0.0231 | 0.0271 |  |  |  |  |  |
|  | Isoleucine | H | 6.75A | 7.27A | 4.86B | 0.4634 | 0.0233 | 0.0459 | 0.0148 | 0.0454 |
|  |  | L | 6.90A | 5.90A | -13.2B | 4.9275 | 0.0476 |  |  |  |
|  |  | SEM | 0.5012 | 0.5336 | 6.017 |  |  |  |  |  |
|  |  | *P*-value | 0.8410 | 0.1434 | 0.1013 |  |  |  |  |  |
|  | Methionine | H | 4.15A | 4.31A | 0.895B | 0.4330 | 0.0023 | 0.0024 | < 0.0001 | 0.3213 |
|  |  | L | 3.48A | 2.35A | -0.481B | 0.6374 | 0.0116 |  |  |  |
|  |  | SEM | 0.3310 | 0.5777 | 0.6697 |  |  |  |  |  |
|  |  | *P*-value | 0.2273 | 0.0742 | 0.2179 |  |  |  |  |  |
|  | Threonine | H | 3.27 | 3.27 | -5.77 | 4.063 | 0.2683 | 0.6948 | 0.0640 | 0.9647 |
|  |  | L | 3.08 | 1.75 | -8.21 | 3.8772 | 0.1594 |  |  |  |
|  |  | SEM | 0.5049 | 0.7124 | 6.823 |  |  |  |  |  |
|  |  | *P*-value | 0.7997 | 0.2050 | 0.8128 |  |  |  |  |  |
| Nonessential amino acids | Alanine | H | 8.20A | 8.72A | 6.36aB | 0.4695 | 0.0271 | 0.0338 | 0.0003 | 0.1430 |
|  |  | L | 8.41A | 7.43A | 4.46bB | 0.5048 | 0.0036 |  |  |  |
|  |  | SEM | 0.5279 | 0.4850 | 0.4459 |  |  |  |  |  |
|  |  | *P*-value | 0.7995 | 0.1337 | 0.0396 |  |  |  |  |  |
|  | 4-Aminobutyric acid | H | 8.10A | 8.39A | 5.89B | 0.3959 | 0.0081 | 0.0245 | < 0.0001 | 0.2586 |
|  |  | L | 8.02A | 7.14A | 4.36B | 0.5010 | 0.0050 |  |  |  |
|  |  | SEM | 0.4783 | 0.4635 | 0.0496 |  |  |  |  |  |
|  |  | *P*-value | 0.9182 | 0.1296 | 0.0578 |  |  |  |  |  |
|  | Proline | H | 7.26A | 7.76A | 5.00B | 0.4606 | 0.0117 | 0.0340 | < 0.0001 | 0.0880 |
|  |  | L | 7.66A | 6.37A | 2.77B | 0.7482 | 0.0090 |  |  |  |
|  |  | SEM | 0.4113 | 0.7075 | 0.6985 |  |  |  |  |  |
|  |  | *P*-value | 0.5367 | 0.2369 | 0.0873 |  |  |  |  |  |
|  | Serine | H | 7.31A | 7.79A | 5.27B | 0.4272 | 0.0128 | 0.0365 | 0.1118 | 0.2133 |
|  |  | L | 7.21 | -2.09 | -13.0 | 7.1394 | 0.2143 |  |  |  |
|  |  | SEM | 0.5069 | 6.224 | 6.137 |  |  |  |  |  |
|  |  | *P*-value | 0.8951 | 0.3243 | 0.1029 |  |  |  |  |  |
|  | Glycine | H | 5.79A | 6.19A | 3.87B | 0.4583 | 0.0247 | 0.0383 | 0.0151 | 0.0479 |
|  |  | L | 5.61A | 4.49A | -13.6B | 4.7099 | 0.0485 |  |  |  |
|  |  | SEM | 0.5281 | 0.5757 | 5.742 |  |  |  |  |  |
|  |  | *P*-value | 0.8305 | 0.1054 | 0.0982 |  |  |  |  |  |
|  | L-Allothreonine | H | 5.02A | 5.56A | 3.01B | 0.4482 | 0.0158 | 0.0369 | 0.1041 | 0.2100 |
|  |  | L | 5.02 | -3.52 | -13.8 | 6.5394 | 0.2073 |  |  |  |
|  |  | SEM | 0.5153 | 5.721 | 5.608 |  |  |  |  |  |
|  |  | *P*-value | 0.9969 | 0.3246 | 0.1019 |  |  |  |  |  |
|  | Tyrosine | H | 3.53A | 4.04A | 1.82B | 0.4877 | 0.0412 | 0.4643 | 0.0003 | 0.1153 |
|  |  | L | 4.42A | 3.62A | 0.429B | 0.4853 | 0.0026 |  |  |  |
|  |  | SEM | 0.5250 | 0.4360 | 0.4943 |  |  |  |  |  |
|  |  | *P*-value | 0.2965 | 0.5298 | 0.1171 |  |  |  |  |  |
|  | Aspartic acid | H | 3.45A | 3.53aA | 0.458B | 0.3515 | 0.0013 | 0.0073 | 0.0001 | 0.5714 |
|  |  | L | 2.64A | 1.63bA | -0.935B | 0.6772 | 0.0240 |  |  |  |
|  |  | SEM | 0.4444 | 0.4762 | 0.6699 |  |  |  |  |  |
|  |  | *P*-value | 0.2659 | 0.0482 | 0.2154 |  |  |  |  |  |
|  | Ornithine | H | 2.66 | 3.11a | 1.01a | 0.6431 | 0.1268 | 0.0001 | 0.0010 | 0.1835 |
|  |  | L | 1.09A | 0.168bA | -2.80bB | 0.3844 | 0.0009 |  |  |  |
|  |  | SEM | 0.7167 | 0.3809 | 0.4280 |  |  |  |  |  |
|  |  | *P*-value | 0.1957 | 0.0054 | 0.0033 |  |  |  |  |  |
|  | Beta-Alanine | H | -5.43 | 1.04 | -1.37 | 3.781 | 0.5126 | 0.7844 | 0.6201 | 0.2956 |
|  |  | L | 0.430A | -1.11AB | -3.20B | 0.7076 | 0.0302 |  |  |  |
|  |  | SEM | 4.631 | 0.7101 | 0.5009 |  |  |  |  |  |
|  |  | *P*-value | 0.4214 | 0.0987 | 0.0613 |  |  |  |  |  |
|  | Asparagine | H | -5.42 | -5.19 | -19.8a | 5.331 | 0.1666 | 0.6972 | 0.0124 | 0.4461 |
|  |  | L | -0.660A | -13.0B | -21.4bB | 3.4502 | 0.0152 |  |  |  |
|  |  | SEM | 4.634 | 6.239 | 0.2882 |  |  |  |  |  |
|  |  | *P*-value | 0.5079 | 0.4263 | 0.0167 |  |  |  |  |  |
|  | Glutamic acid | H | -0.156A | 0.370aA | -2.62B | 0.2770 | 0.0006 | 0.0068 | < 0.0001 | 0.2062 |
|  |  | L | -0.482A | -1.06bA | -3.30B | 0.2572 | 0.0006 |  |  |  |
|  |  | SEM | 0.3227 | 0.1043 | 0.3150 |  |  |  |  |  |
|  |  | *P*-value | 0.5142 | 0.0006 | 0.2010 |  |  |  |  |  |

^1^ Values with different lowercase letters (a and b) indicate significant differences among treatments on the same day during aerobic exposure.

^2^ Values with different uppercase letters (A, B, and C) indicate significant differences among days after opening for the same treatments (*P* < 0.05).

^3^ H, ensiled whole corn plants with a high moisture content (680 g/kg) harvested at the one-third milk-line stage; L, ensiled whole corn plants with a low moisture content (630 g/kg) harvested at the two-thirds milk-line stage.

^4^ SEM, standard error of means.

^5^ M, moisture (silages with high or low moisture contents); T, aerobic exposure time; M × T, interaction between the moisture content and the aerobic exposure time.
